# Does the timing of breast cancer surgery in pre-menopausal women affect clinical outcome? : an update

**DOI:** 10.1186/1477-7800-3-37

**Published:** 2006-11-01

**Authors:** Anushka Chaudhry, Michael L Puntis, Panos Gikas, Kefah Mokbel

**Affiliations:** 1 Dept. of Breast Surgery, St George's Hospital, University of London, Tooting, UK; 2 The Princess Grace Hospitals, 42-52 Nottingham Place, London W1M 3FD, UK

## Abstract

There is some evidence that breast cancer surgery during the luteal phase in pre-menopausal women is associated with a better clinical outcome, however the evidence for this is still equivocal.

In this paper, after summarizing the normal physiology of the menstrual cycle, we examine how such an association may occur and provide a comprehensive review of the literature in the area.

## Background

Breast cancer is a significant disease affecting over 41,000 women each year in the UK [1]. One in three of these patients are younger than 55 when the diagnosis of breast cancer is made, and almost all will undergo wide local excision or mastectomy.

Amongst the significant sub-group of pre-menopausal patients the tumour is likely to be subject to cyclical fluctuations in the hormonal milieu. Any impact on survival from the coordination of surgery with the phase of the menstrual cycle should therefore be explored and changes to clinical practice could be implemented as indicated.

There is some evidence that breast surgery during the luteal phase in pre-menopausal women is associated with better outcomes, however the evidence for this is not consistent (see table 1)

**Table 1:** Summary of key studies

Year	Author	Study Design	Outcome measure	Outcome difference	P value
2003	Takeda [31]	Retrospective n = 36	Disease-free survival	Nil	n/a
2002	Fentiman [29]	Prospective n = 249	10 year survival	84% luteal 54% follicular	-
2001	Pujol [30]	Prospective n = 360	Disease-free survival	nil	n/a
1999	Nomura [32]	Retrospective n = 721	Disease-free survival	nil	n/a
1998	Mangia [33]	Retrospective N = 248	Disease-free survival	Nil	n/a
1998	Harlap [34]	N = 614	10-year survival	nil	n/a
1997	Goldhirsch [35]	RCT n = 300	Disease-free survival (ER-ve)	59% luteal 42% follicular	P = 0.008
1997	Vanek [36]	Retrospective	Overall Survival	Nil	n/a
1997	Mondini [37]	N = 165	Overall Survival	Nil	n/a
1995	Jager [38]	Retrospective n = ?	Disease-free survival	nil	n/a
1995	Holli [39]	N = 267	Overall Survival	nil	n/a
1994	Kroman [40]	N = 1635	Overall Survival	nil	n/a
1994	Corder [41]	N = 157	Overall Survival	nil	n/a
1994	Saad [42]	Retrospective N = 96	Overall Survival	79% lut 40% foll,	P < 0.001
1992	Gnant [43]	Retrospective n = 385	Overall Survival	nil	n/a
1991	Badwe [27]	Retrospective n = 75	Overall survival	54% follicular 84% luteal	P < 0.001
1991	Senie [44]	Prospective n = 283	Disease-free survival	71% luteal 57% follicular	P = 0.02
1989	Hrushesky [7]	Retrospective N = 44	Overall Survival	95% luteal 79% follicular	-

Although there are data suggesting that hormonal factors influence tumour characteristics and behaviour, however there is no definitive evidence that surgery should be scheduled to avoid the follicular phase. A number of studies have investigated the biochemical and clinical response of tumours resected during the various phases of the menstrual cycle [2-4]. In addition, the hormonal status of a patient may have an impact on intra-operative factors such as bleeding [5], and may be also significant for the timing of core biopsies [6].

In this paper, after summarizing the normal physiology of the menstrual cycle, we examine how such an association may occur and provide a comprehensive review of the literature in the area.

### Normal physiology

The menstrual cycle is a rhythmic preparation for extrusion of an ovum and subsequent pregnancy if the ovum is fertilized. The events are under the influence of the hypothalamic gonadotrophins follicle stimulating hormone (FSH) and luteinising hormone (LH) that regulate the release of sex steroid hormones estrogen and progesterone from the ovarian interstitium. These in turn prime the ovarian follicles and induce ovulation. Assuming that Day 0 is the first day of menstrual flow of the last menstrual period, all hormones are low for the first 4–5 days. This is the early period of follicular development. Estrogens gradually rise for the next 3–4 days followed by a rapid peak by 12^th ^day which is the day before the LH and FSH peak. Ovulation then occurs 24–36 h after the LH peak by about the 14^th ^day. The second peak of estrogen occurs about a week after ovulation and is opposed by a progesterone peak. Furthermore, progesterone shows a small increase in concentration corresponding to the LH surge. Hence estrogen remains unopposed during the follicular phase, up to Day 12, while during the rest of the cycle progesterone opposes the action of estrogen.

### The hypotheses

In 1989 Hrushesky et al. were the first to propose that premenopausal patients with breast cancer who underwent surgery during the luteal phase of the menstrual cycle had higher disease-free and overall survival rates than did patients operated on during other phases of the cycle [7]. It is indeed an intriguing aspect of the treatment of breast cancer that the timing of breast surgery, in relation to menstrual cycle phase and hence hormonal status, might influence the natural history and prognosis of the disease. The biological-endocrinological basis for this is two-fold:

Firstly, in the follicular phase, estrogens reduce immune activity, phagocytic activity and circulating levels of IL-2 therefore potentially increasing metastatic potential of breast cancer cells [8]. At the same time they stimulate the activity of insulin-like growth factor (IGF), which has been proven in several studies to have an important mitogenic effect on breast cells [9-11]. Furthermore, angiogenesis, a process vital for the progression of many tumours and hence for their ability to metastasise, is favoured by high circulating levels of estrogens since they promote expression of vascular endothelial growth factor (VEGF) [12,13].

Secondly, in the luteal phase the increase in circulating levels of endogenous progesterone may modulate the proliferation of normal and neoplastic breast tissue and increase intercellular cohesion thereby reducing metastatic potential [14,15]. Progesterone also acts to regulate the IGF1 (pro-mitotic effect)/TGFb (anti-mitotic effect) balance. Moreover progestogens act to reduce the number of estrogen receptors expressed on breast cells hence limiting estrogenic stimulation and as a result promoting breast cell apoptosis [16].

Excision of tumours in the presence of unopposed oestrogen has been reported to be associated with improved survival. This was initially suggested by the better outcomes of peri- and post-menopausal patients. Thin patients, with less peripheral aromatase, have lower oestrogen compared to obese patients [17]. An improved outcome in these groups has been demonstrated in meta-analysis in both pre and post menopausal patients [18] but this does not prove causation. Furthermore, many patients in these groups may have compounding factors causing the variation in survival.

It has been demonstrated that there is a reduced breast tumour growth during the pre or peri-ovulatory phases, but the tumour then increases in size during the post-ovulatory phase, before shrinking back towards the original dimension. In animal studies, mice with faster cycles have slower tumour growth rates. This correlation has even been demonstrated in animal models of non-hormone dependant tumours (sarcoma) [19].

### Increased adrenergic immunosuppression

During the menstrual cycle, fluctuation in hormone levels produce a modulation of adrenergic mechanisms and through this NK cells responsible for metastasis control become suppressed secondary to increased adrenergic tone during the follicular phase [20]. Animal studies have also confirmed this correlation between NK metastasis suppression and adrenergic modulation during oestrous, which can be increased with an exogenous beta agonist [21].

### Vascular endothelial growth factor

In the female reproductive system angiogenesis occurs as a normal process and is essential for normal tissue development. In the ovary new blood vessel formation facilitates oxygen and nutrient delivery and allows the transfer of hormones to targeted cells, ovarian follicles and the corpus luteum have been shown to produce several angiogenic factors, of which vascular endothelial growth factor (VEGF) is thought to be paramount. VEGF levels fluctutate within the breast with greater concentrations occurring during the luteal phase [22], interestingly there is no evidence for a corresponding fluctuation in serum levels [23]. This modulation of proangiogenic cytokines in the breast may contribute to the reduced potential for metastasis in tumours resected during the luteal phase [22-24].

### Tumour epidermal growth factor

A prospective study of premenopaual women measured the concentrations of oestrogen, porlaction, progesterone, tumour epidermal growth factor and tumour epidermal growth factor receptor. While levels of tumour epidermal growth factor remained constant EGFR levels peaked during the follicular phase [25]. This cyclical up-regulation may contribute to the worse prognosis potentially associated with surgery during this phase. Other studies have demonstrated that the EGF receptor is localised in the epithelial cells during the luteal phase [26].

### Other factors

Surgery for benign disease such as mammaplasty, associated with reduced intraoperative bleeding and avoidance of a post-operative drain if carried out during the periovulatory phase [5]. Increased tumour vascular invasion and Her-2 expression during the follicular phase ans regulation of matrix metallo-proteinases (MMP 2 & 9) and MMP tissue inhibitors (TIMP-1 & 2) are also potential factors [27-29].

### Review of the literature

In the following paragraphs we will attempt to review the literature, so far published on this intriguing hypothesis, and therefore identify the clinical implications that such a hypothesis has in relation to the treatment of breast cancer. There has been no comprehensive systematic review of literature since 2001, despite recent published evidence in this area.

The relationship between the menstrual cycle and surgical outcome was first described by Hrushesky in 1989 [7]. Based on a retrospective study of 44 patients, it was postulated that surgery carried out on premenopausal patients during the luteal phase confers higher disease free survival (DFS) compared to patients who underwent surgery at other times.

In 1991 Badwe et al [27], suggested that unopposed oestrogen may be associated with increased mortality, with a 10 year survival rate of 54% in the patients whose surgery took place during days 2–13, compared to 84% in those undergoing surgery between days 0–2 and 13–32.

A large meta-analysis using a fixed effect model of 37 published studies (n = 10476) was conducted in 2000 [28] and demonstrated a 15% (+/- 4%) increase in survival when tumours were resected during the luteal phase (p = 0.003), however three further studies [29-31] have been carried out since then, adding significantly to the body of evidence in this field.

In 2002, a retrospective analysis of prospectively collected data and serum samples from 249 pre-menopausal patients undergoing resection of breast tumours showed that in both node positive and node negative tumours, the 10 year survival is improved by carrying out surgery during the luteal phase which was defined by both last menstrual period (LMP) and serum progesterone [29]. The 10-year survival for node positive cases undergoing follicular phase surgery was 33% compared with 78% in those having surgery at other times of the menstrual cycle.

Conversely, a prospective study of 360 pre-menopausal patients, published in 2001, using hormonal assays to time the menstrual phase demonstrated no prognostic influence of coordinating surgery with the luteal phase [30]. Furthermore, a more recent small study of 36 patients showed that the recurrence rate and relapse-free survival were not significantly different with the menstrual timing of surgery [31]. However, patients with early breast cancer undergoing surgery during the follicular phase and those with advanced breast cancer resected during the luteal phase appeared to show better prognosis than corresponding controls operated during the other phases.

Table 1 summarises the key studies in this field including several studies [32-44] already used in the 2000 meta-analysis [28].

## Discussion

There is clearly a controversy surrounding the most appropriate timing of breast cancer surgery and there is inconsistent evidence supporting the notion that resection during the luteal phase improves outcome. Twenty-two out of the 40 studies published were in favour of luteal phase surgery compared with 9 studies in favour of follicular phase surgery [28]. The remainder of studies (n = 9) showing no significant difference between the two phases. However breast cancer amongst pre-menopausal women is such a critical topic that even modest advantages in outcome should be exploited.

Many of the initial studies in this field were undertaken retrospectively and often relied on inaccurate assessment of the hormonal status of the patient. Using the LMP to define the cycle phase is thought to be inaccurate since factors such as peri-operative stress and the impact of a recent diagnosis of breast cancer may result in disruption of the normal menstrual cycle or even an anovulatory cycle. However recent studies have been conducted prospectively and with sound methodology, relying on serum hormone analysis to define the menstrual phase in addition to the LMP and have added significantly to the available data regarding the appropriate timing of breast surgery.

There is still discordance between studies and well-conducted prospective studies have provided evidence, both for and against, rescheduling surgery to the luteal phase. Meta-analysis [28] has demonstrated that luteal phase surgery leads to improved survival and this policy has been already adopted in some centres especially in node positive, and hormone receptor positive tumours. However this meta-analysis should be interpreted with extreme caution since it has significant inherent limitations including the presence of significant heterogeneity between studies, publication bias, the lack of defining criteria for study inclusion and the lack of adjustments for confounders and covariates.

Rescheduling surgery to coincide with the luteal phase would appear to be a simple intervention that can be considered in order to improve outcome, however there are further considerations. If scheduling surgery is dependant on the hormonal status of the patient then the available window for surgery becomes more limited, which will place increased logistical demands on the unit and surgeons performing breast cancer surgery, it is even possible that surgery may have to be delayed while waiting for the appropriate phase.

While studies vary in their conclusions, it may be prudent to defer the large scale rescheduling of breast cancer surgery until a sound biological hypothesis is defined, and corroborated by ongoing randomised controlled trials that use biochemical testing to accurately define the cycle phase. Furthermore, trials examining the potential role of neo-adjuvant endocrine therapy to counteract unopposed oestrogen during the follicular phase are required.

In addition to the cyclical variation in cell cohesiveness, growth receptors and angiogenesis in tumours, there are a number of hypotheses that may account for the potential difference in survival. This underscores the need for further studies aiming to define which specific mechanisms may be responsible and such research will enhance our understanding of mammary carcinogenesis.
